# Control of Cytokines in Latent Cytomegalovirus Infection

**DOI:** 10.3390/pathogens9100858

**Published:** 2020-10-21

**Authors:** Pearley Chinta, Erica C. Garcia, Kiran Hina Tajuddin, Naomi Akhidenor, Allyson Davis, Lionel Faure, Juliet V. Spencer

**Affiliations:** Department of Biology, Texas Woman’s University, P.O. Box 425799, 1000 Old Main Circle, Denton, TX 76204, USA; pchinta@twu.edu (P.C.); egarcia48@twu.edu (E.C.G.); ktajuddin@twu.edu (K.H.T.); nakhidenor@twu.edu (N.A.); adavis66@twu.edu (A.D.); lfaure@twu.edu (L.F.)

**Keywords:** chemokines, cmvIL-10, cytokines, cytomegalovirus, interferons, ISGs, latency

## Abstract

Human cytomegalovirus (HCMV) has evolved a number of mechanisms for long-term co-existence within its host. HCMV infects a wide range of cell types, including fibroblasts, epithelial cells, monocytes, macrophages, dendritic cells, and myeloid progenitor cells. Lytic infection, with the production of infectious progeny virions, occurs in differentiated cell types, while undifferentiated myeloid precursor cells are the primary site of latent infection. The outcome of HCMV infection depends partly on the cell type and differentiation state but is also influenced by the composition of the immune environment. In this review, we discuss the role of early interactions between HCMV and the host immune system, particularly cytokine and chemokine networks, that facilitate the establishment of lifelong latent infection. A better understanding of these cytokine signaling pathways could lead to novel therapeutic targets that might prevent latency or eradicate latently infected cells.

## 1. Introduction

Human cytomegalovirus (HCMV) is a member of the *Herpesviridae* family that is widespread in the general population. HCMV is transmitted via contact with body fluids, including blood, semen, breast milk, urine, and saliva. Infection is typically mild or asymptomatic, and nearly half the population of the United States is seropositive for HCMV by age five [1]. Despite a vigorous immune response to HCMV, the virus is never cleared and instead establishes lifelong latent infection in the host [2]. Latency is a dormant state characterized by limited expression of viral genes without viral DNA replication or production of infectious viral progeny. Periodic reactivation of lytic virus replication occurs routinely even in healthy immunocompetent individuals, which contributes to widespread transmission. Usually, reactivation is limited by host immune responses, however, clinical reactivation events may occur in the immunosuppressed. HCMV disease caused by reactivation of the latent virus is one of the leading complications following solid organ and hematopoietic stem cell transplants [3]. While antiviral drugs like ganciclovir are available for treatment, patient outcomes are hindered by drug toxicity, emerging virus strains with drug resistance, and the inability of the antivirals to target latently infected cells.

Primary HCMV infection usually occurs in epithelial cells at the mucous membranes of the oropharynx, while latency is established in CD34^+^ myeloid progenitor cells [2]. In these undifferentiated cells, the lytic replication cycle is prevented by suppressing the transcriptional activity of the Major Immediate Early Promoter (MIEP). As CD34^+^ cells differentiate into macrophages and dendritic cells, Immediate Early (IE) genes are expressed, and lytic replication ensues. Like all viruses, HCMV induces an antiviral host immune response mediated by NK cells, B cells, and T cells, and coordinated by cytokines [4]. Cytokines are small extracellular proteins that facilitate communication between immune cells, regulating their growth, maturation, and response to infection [5]. The large cytokine family has been divided into groups that include interferons (IFN), interleukins (IL), tumor necrosis factors (TNF), and chemokines. The roles of representative members of each group in HCMV infection and latency are described below.

### 1.1. Interferons

IFNs are a pivotal group of cytokines that influence the outcome of virus infection, and they are named for their ability to interfere with viral replication. There are three types of IFNs: Type I, Type II, and Type III, and these are distinguished by the cellular receptor to which they bind [6]. When a cell becomes infected with a virus, the cell responds by producing Type I IFNs, mainly IFNα and IFNβ, which bind to neighboring cells to induce an antiviral state. Type II IFNs (IFNγ) are produced mainly by immune cells and promote antigen presentation [7]. IFNs induce expression of a cascade of genes known as Interferon-Stimulated Genes, or ISGs, that limit virus replication.

The most successful viruses, including HCMV, have evolved ways to counteract IFN signaling [7,8]. In particular, the HCMV IE proteins, IE1 (p72, UL123) and IE2 (p86, UL122), are two negative regulators of the IFNβ pathway [9,10]. IE1 binds to Signal Transducer and Activator of Transcription 1 and 2 (STAT1/2) and IFN-Regulatory Factor 9 (IRF9), thereby preventing these regulators from binding to the IFN-Stimulated Response Element (ISRE) that is present in the promoter region of most ISGs [10]. The result is reduced ISG expression and attenuation of the innate antiviral immune response. In contrast, IE2 blocks ISGs through antagonism of Nuclear Factor Kappa B (NF-κB) DNA binding activity [11]. NF-κB is a master regulator of innate immunity, and interference with NF-κB not only reduces ISG levels but also suppresses the expression of pro-inflammatory cytokines IL-1, IL-6, TNFα, as well as IFNβ itself [12,13,14,15,16,17]. IE1 and IE2 are critical factors in lytic virus replication, and expression of these genes is regulated by the powerful MIEP. As noted, suppression of the MIEP is necessary to block the lytic cascade and allow the establishment of HCMV latency in myeloid cells. One factor known to mediate MIEP repression is US28 [18], a viral chemokine receptor that also suppresses the expression of ISGs during latency [19] (discussed below). Conversely, IE1 and IE2 play a role in thwarting IFNs early in lytic infection, which helps to delay the host antiviral immune response, allowing time for the establishment of latency and facilitating immune escape during reactivation.

In contrast to IE proteins that require gene expression and protein synthesis following virus infection, tegument proteins enter with the virus particle and can immediately influence host cell physiology. Two tegument proteins have been found to inhibit interferon responses and ISG expression: UL26 and pp71 (UL82). The pp71 protein inhibits IFNα by blocking the Stimulator of Interferon Genes (STING) [20]. Cells normally detect viral DNA through the DNA-binding protein cyclic GMP-AMP Synthase (cGAS), which produces cGAMP upon binding to DNA in the cytoplasm. The cGAS-STING pathway is activated when cGAMP binds to STING, leading to phosphorylation of Interferon Response Factor 3 (IRF3), which stimulates expression of type I IFNs. Pp71 inhibits cellular trafficking of STING and disrupts complex formation, directly reducing the expression of IFNα.

Upon virus entry, the tegument protein UL26 localizes to the nucleus where it helps stimulate expression from the MIEP and antagonizes host NF-κB activity [21]. Infection with a UL26 deletion virus results in higher expression of both NF-κB target genes and ISGs, including ISG15 and bone marrow stromal cell antigen 2 (BST2) [22]. While no specific role in latency has been defined, UL26 plays an important role in protecting HCMV from innate antiviral responses so that latency can be established. Both pp71 and US26 contribute to dampening the initial interferon-mediated antiviral response to HCMV.

### 1.2. Interleukins and Tumor Necrosis Factor

There are more than fifty different interleukins (ILs), a group of cytokines that were initially defined by the fact that they were secreted from white blood cells. The interleukin-1 (IL-1) family of cytokines triggers innate inflammation in the presence of viruses, bacteria, Damage-Associated Molecular Patterns (DAMPSs), and microbial products [23,24]. IL-1 and IL-6, along with TNFα, are the primary mediators of acute, pro-inflammatory responses during HCMV infection. The TNF family of cytokines bind to distinct receptors from the ILs, and play roles not only in inflammation, but also in the regulation of apoptosis [20,24]. Many viral proteins that block IFN activity also suppress the expression of IL-1, IL-6, and TNFα, such as IE1, IE2, pp71, and UL26, further impairing the initial inflammatory response.

In addition to viral proteins, HCMV also uses microRNA (miRNA) to reduce the expression of pro-inflammatory cytokines IL-1β, IL-6 and TNFα. In particular, miR-US5-1 and miR-UL112-3p are present in the HCMV particle and block NF-κB signaling. They reduce the expression of IκB kinase-α and β (IKKα/β), thereby preventing the degradation of IκB and limiting cytokine production [25]. By maintaining a stock of this miRNA during latency, the virus creates an environment that ensures its survival in the host. Many miRNAs are expressed by HCMV [26], which could represent a strategy for regulating cellular gene expressions to promote latency without viral proteins that could trigger antiviral immune responses.

The UL144 gene of HCMV encodes a protein with similarity to members of the TNF superfamily [27,28]. To date, no TNF ligand has been found to interact with the UL144 receptor [29], making it unlikely that this receptor acts as a sponge to soak up TNFα and prevent an inflammatory response. However, unlike IE2 and UL26, which block NF-κB activation, UL144 stimulates the NF-κB pathway. When expressed in U-373 glioblastoma cells, UL144 caused NF-κB activation that was dependent on TNF Receptor Activated Factor 6 (TRAF6) and Tripartite Motif 23 protein (TRIM23) [30]. One particular NF-κB target gene that was upregulated was the chemokine CCL22, which attracts cells expressing the receptor CCR4. Production of CCL22 could aid in immune evasion by skewing toward a Th2 type response, which is tailored to parasites and intestinal helminths and is less effective against viral infections.

The UL138 gene is considered a key factor in the establishment of HCMV latency [31,32]. Originally identified as a Golgi-localizing factor, the UL138 protein was shown to upregulate surface expression of TNFα-receptor 1 (TNFR-1), a phenomenon not observed during infection with virus strains that lack the ULb’ region where the UL138 locus is found [33,34]. The observation that UL138 enhances TNFR-1 levels has led to speculation that latently infected cells might then become more sensitive to TNFα, potentially leading to lytic reactivation [33]. A different role for UL138 involves interaction with UL133, UL135 and UL136 gene products to form a protein complex that reduces lytic virus replication and promotes latency in human CD34^+^ myeloid progenitor cells [31,32,35,36,37]. UL138 has also been found to affect surface levels of Epidermal Growth Factor Receptor (EGFR), with increased signaling activity enhancing latent infection of CD34^+^ myeloid progenitor cells [38,39]. EGFR signaling upon infection of CD34^+^ cells impacts the expression of several cytokines, including IL-8 and IL-12 [39]. It is not yet clear how these cytokines promote HCMV latency, and further investigation of the role of the UL138 protein is needed.

### 1.3. Interleukin 10

Interleukin 10 (IL-10) is one of the most important regulators of the immune system. IL-10 suppresses and controls the magnitude of inflammatory responses, and in doing so, has the ability to limit virus clearance and promote virus persistence [40,41,42]. The UL111A gene of HCMV encodes an ortholog of human IL-10 (hIL-10), known as cmvIL-10. UL111A is expressed with late kinetics during lytic infection, and cmvIL-10 is secreted as a dimer from infected cells. Although sequence identity between cmvIL-10 and hIL-10 is low, the tertiary structure is similar, allowing cmvIL-10 to bind the cellular IL-10 receptor, activate transcription factor STAT3, and share many functional similarities with hIL-10 [43,44,45]. Through cmvIL-10, HCMV prevents the production of pro-inflammatory cytokines IL-1α, GM-CSF, IL-6, and TNF-α by monocytes and dendritic cells and also decreases levels of both Major Histocompatibility Complex (MHC) I and II [44,46]. In sum, the effects of cmvIL-10 suppress T cell activation, which impedes virus clearance and enables latent infection of myeloid progenitor cells.

A particular isoform of cmvIL-10 that is important in latency is latency-associated cmvIL-10, or LAcmvIL-10 [47]. The UL111A gene contains three exons and two introns [48]. The full-length cmvIL-10 protein is 175 amino acids in length and produced when both introns are spliced; however, alternative splicing can lead to retention of intron 2, which contains a stop codon [47]. This spliced transcript produces LAcmvIL10, a protein that is 139 amino acids long [47]. LAcmvIL-10 is collinear with cmvIL-10 for the first 127 amino acids and diverges in the 12 amino acids at the C-terminus. While cmvIL-10 is glycosylated, LAcmvIL-10 is not due to the absence of the N151 glycosylation site in the C-terminal domain [49]. These differences alter the signaling ability of LAcmvIL-10 and ultimately restrict the set of functions possessed by this cytokine. LAcmvIL-10 does not appear to interact with IL-10R, nor does it activate STAT3 [50]. This is not to suggest that LAcmvIL-10 cannot modify the host environment. During latency, LAcmvIL-10 suppresses the cellular microRNA hsa-miR-92a, leading to the upregulation of chemokine CCL8. CCL8, also known as Monocyte Chemoattractant Protein 2 (MCP-2) attracts both monocytes and CD4^+^ T cells. CD4^+^ T cells are rendered ineffective at cell killing in the presence of factors like LAcmvIL-10 that are secreted from cells infected with HCMV [51], thus ensuring their survival.

In addition to CCL8, the downregulation of hsa-miR-92a by LAcmvIL-10 also leads to the upregulation of hIL-10 secreted by CD34^+^ myeloid progenitor cells and CD14^+^ macrophages [52]. During latency, the induction of hIL-10 suppresses inflammatory cytokines and impairs T cell effector functions, allowing infected cells to escape immune detection. Stimulation of hIL-10 is significant because LAcmvIL-10 lacks the ability to engage the IL-10R, so the cellular cytokine is recruited to provide additional immune suppression for the maintenance of HCMV latency.

Interestingly, LAcmvIL-10 is produced not only during latency, but also during lytic infection [49]. Together, cmvIL-10 and LAcmvIL-10 have a broad range of powerful effects, particularly the ability to induce hIL-10. While LAcmvIL-10 works through hsa-miR-92, cmvIL-10 acts by upregulating the positive regulatory factor Tumor Progression Locus 2 (TPL2), a member of the Mitogen-Activated Protein Kinase (MAPK) signaling cascade [53]. CmvIL-10 upregulates Heme Oxygenase-1 (HO-1), which is required for the upregulation of hIL-10. HO-1 also plays a role in the suppression of the inflammatory response [53]. Though cmvIL-10 is produced during the lytic infection, it is an important component of the anti-inflammatory and immune evasive strategies possessed by HCMV. Little is known about the regulation of splicing and expression from the UL111A locus, and more work is necessary to establish how cmvIL-10 contributes to HCMV latency. In combination, these effects of cmvIL-10, LAcmvIL-10, and amplification of hIL-10 are key for maintaining latency and avoiding detection by the host immune system.

### 1.4. Chemokines

Many viruses exploit chemokine signaling to benefit their life cycle [54]. Chemokines are chemotactic cytokines, and they induce directed cell movement, or chemotaxis, upon receptor binding. Chemokines are grouped into four subfamilies based on the number and spacing of conserved cysteine residues [55]. These sub-families include C-chemokines (e.g., XCL1 or lymphotactin-α), CC-chemokines (e.g., CCL5 or RANTES), CXC-chemokines (e.g., CXCL1 or GROα), and CX_3_C chemokines (CX_3_CL1, or fractalkine is the sole member of this group) [56]. HCMV encodes two genes, UL146 and UL147, which give rise to CXC chemokines, designated vCXCL1 and vCXCL2, respectively. vCXCL1 binds and signals through the human chemokine receptors CXCR1 and CXCR2, inducing calcium mobilization and chemotaxis of neutrophils [57,58]. In addition, vCXCL1 also binds to CX_3_CR1 to induce natural killer (NK) cell migration [59]. While neutrophils are considered a vehicle for virus dissemination throughout the host, it is not clear why HCMV would deliberately attract NK cells that could eliminate virus-infected cells. However, HCMV does have many mechanisms for evading NK cell detection [60], and it may be that vCXCL1 is less effective at attracting NK cells in vivo. Even in vitro, vCXCL1 preferentially bound to CXCR2 to attract neutrophils more rapidly and efficiently than NK cells [59]. This experimental result highlights the exquisite control that HCMV exhibits in the manipulation of host immune responses. Although UL146 and UL147 are expressed with late kinetics during lytic infection, recent evidence suggests that both genes are also expressed at low levels during latent infection of CD34^+^ progenitor cells [61]. The role of these viral chemokines in latency remains to be determined.

The UL128 gene of HCMV encodes a CC chemokine [62,63]. The UL128 gene product (pUL128) is a member of the pentameric complex that facilitates virus entry into epithelial and endothelial cells [64]. HCMV glycoproteins gH and gL mediate attachment and entry, but they do so in a complex with either glycoprotein gO, forming a trimeric complex (gH/gL/gO), or in a pentameric complex with pUL128, pUL130 and pUL131A (gH/gL/pUL128/pUL130/pUL131A) [65]. The pentameric complex facilitates virus entry into the monocytes by activating integrin-mediated signaling [66]. pUL128 is located at the tip of the pentameric complex and binds non-covalently to pUL131A [64]. As part of the pentameric complex, pUL128 impairs monocyte migration by reducing surface levels of chemokine receptors CCR1, CCR2, and CCR5 [63]. In contrast, pUL128 recruits peripheral blood mononuclear cells (PBMCs) and promotes the expression of TNFα and IL-6 via activation of the MAPK pathway [67]. Further investigation is required to understand the roles of pUL128 in HCMV infection beyond virus entry. To date, the UL128 gene has not been found to be expressed during latency [61], suggesting that any chemokine signaling contributes to immune modulation mainly during lytic infection.

In addition to chemokines, HCMV also encodes four genes whose gene products resemble human chemokine receptors: US27, US28, UL33, and UL78. Chemokine receptors are seven-transmembrane domain receptors that interact with heterotrimeric G proteins for downstream signaling. Chemokine receptors are one class of the G protein-coupled receptor (GPCR) superfamily. While most GPCRs require ligand binding for activation, the HCMV GPCRs are constitutively active and can induce signaling in a ligand-independent fashion [68].

Among the four viral GPCRs (vGPCRs), US28 is the most studied and is expressed during lytic infection with early kinetics and also during latent infection [69]. US28 not only signals constitutively but also in response to several human chemokines, including CCL1, CCL5 (RANTES), CCL7, CCL11, CCL13, CCL26, CCL29, and CX_3_CL1. Studies have shown that the US28 gene is important for the maintenance of latent infection in human progenitor cells [18]. In Kasumi-3 cells, an undifferentiated leukemia cell line, and in primary CD34^+^ progenitor cells, a virus lacking US28 was unable to establish latent infection. Instead, transcription from the MIEP was readily detected during infection with a virus lacking US28 [18]. In contrast, CD34^+^ progenitor cells exhibited suppression of MIEP-driven transcription under latent culture conditions when US28 was present. These findings demonstrate that US28 is required for the establishment or maintenance of successful latent infections in culture.

Other studies have detected US28 mRNA in latently detected in the human acute monocytic leukemia cell line (THP-1), peripheral blood monocytes, and myeloid progenitor cells (CD34+) [61,70,71]. To establish latent infection, US28 suppresses the signaling activity of MAPK and NF-κB. However, viruses that lack US28 initiated a lytic infection in infected monocytes by inducing immediate-early (IE) genes that stimulated the production of infectious virions [72]. US28 suppresses MIEP activity via activation of STAT3 to prevent lytic infection and to maintain latency in CD34^+^ progenitor cells and CD14^+^ monocytes [73]. The establishment of latency requires suppression, if not silencing, of the viral genes encoding the major IE transactivators, IE1 and IE2, which drive viral gene expression and productive viral replication [74].

Phylogenetic analyses indicate that US27 and US28 are products of gene duplication and share a common ancestor with human CX_3_CR1 [75]. However, unlike US28, US27 is only expressed in the late phase of lytic infection and it contributes to the extracellular spread of the virus [76]. US27 constitutively activates the transcription factors Nuclear Respiratory Factor 1 and 2 (NRF-1/2) through a pathway involving G_βɣ_ and IhosphoInositide-3 Kinase (PI3K) [77], but this pathway is active during lytic infection only. Of the four HCMV GPCRs, US27 is the only one that does not appear to be expressed during latency [61].

UL33 and UL78 are the other HCMV GPCRs, and they are expressed during the late phase of lytic infection. However, UL33 and UL78 were also detected in latently infected CD34^+^ progenitor cells via RNAseq analysis [61], but their functions in latency remain unknown. Unlike US27 and US28, UL33 and UL78 have homologs present in the genomes of murine CMV (MCMV) and rat CMV (RCMV) that play roles in latency and dissemination in the animal model systems [78]. M33, the MCMV homolog of UL33, is necessary for the efficient establishment of latency in the salivary glands, spleen, and lung in the mouse model [78]. In mice infected with a virus lacking M33, reactivation of the virus was greatly diminished in salivary gland tissue explants [79]. Interestingly, US28 could substitute for M33, and viruses containing US28 in place of M33 were able to establish latency and reactivate the lytic virus. US28 and M33 have similar signaling profiles and both activate downstream transcription factors like NF-κB, Nuclear Factor of Activated T cells (NFAT), and Cyclic AMP Response Element-Binding protein (CREB) [80]. It has not yet been established whether M33, like US28, plays a role in suppression of the MIEP to promote latent infection.

### 1.5. Latency In Vivo

Due to the high species specificity of HCMV, latency has mainly been studied in vitro culture systems [81,82,83]. While these systems provide valuable insights into the molecular processes governing latent infection, they are less informative for understanding the spatial and temporal aspects of the host immune environment. MCMV is a critical tool for observation of the host environment during virus infection. Murine models of CMV infection have been used frequently in studies of the developmental impact of CMV infection on fetal development due to the similarity between the newborn mouse brain and the human fetal brain [84]. Where human and murine models potentially differ is in the location that CMV establishes latency. HCMV has largely been shown to establish latency in hematopoietic and endothelial tissues, while murine models have shown latency established in a wider range of locations including the epithelial cells of the lungs, liver, spleen, salivary glands, kidneys, and hematopoietic bone marrow cells [85,86]. Latent HCMV has been noted mainly in the bone marrow of human hosts, particularly affecting the CD14^+^ monocytes and CD34 ^+^ progenitor cells [87].

In both human hosts and murine models, the fractalkine receptor CX_3_CR1 has been identified as a potential marker for CMV-specific CD8^+^ T cell populations [87]. CD4^+^ T cells kill infected cells in the salivary glands, thereby playing an important role in controlling MCMV infection [88]. Research into the role of the CD4^ +^ T cells as controllers of infection in the fetal mouse brain found that CD4 ^+^ T cells suppress the reactivation of MCMV in latently infected newborn mice [89]. The role of regulatory T cells (T_regs_) has been studied in both primary infections with MCMV and in latency. Intriguingly, the behavior of the T_reg_ cells was dependent upon the organ in which MCMV had established latency. In the spleen, T_reg_ cells promote the latent viral population by MCMV-specific CD4^+^ and CD8^+^ T cells. The opposite was true when latent MCMV populations were established in the salivary glands where T_reg_ cells place significant limitations on the emergence of IL-10-secreting Foxp3-CD4^+^ T cells [90]. CD8^+^ T cells residing in the lymph nodes are considered a protective measure against viral reactivation. Maintenance of the CD8^+^ T cell population over time is dependent upon a latent viral reservoir, found primarily in latently infected non-hematopoietic cells in the lymphoid organs. Tissue-resident memory T cell populations protect against MCMV reactivation in both the brain and spleen, controlling latent MCMV within the host [91]. This result is highlighted by the observation that depletion of these memory T cells resulted in virus reactivation with increased pro-inflammatory responses.

Humanized mice offer a unique tool to study the mechanisms of HCMV latency when they differ from MCMV mechanisms and validate potential HCMV-based vaccine vectors [92]. While research using humanized mouse models is promising, tissue donor consent is still required as is separate ethical approval from a study utilizing traditional murine models. For many reasons, MCMV will always be an important tool for the study of HCMV.

Rhesus cytomegalovirus (RhCMV) naturally infects Rhesus macaques, making this an excellent animal model to study acute and latent CMV infection and aid in the development of vaccines and therapeutics [93]. Similar to HCMV in the human population, RhCMV is also pervasive in Macaque troops. The kinetics of RhCMV dissemination throughout mixed cohorts of uninfected and infected animals is a function of the persistent shedding of the virus in bodily fluids of infected animals [94]. Since they typically live in large troops in the wild, horizontal transmission throughout the troop is a concern amongst Rhesus populations. This poses an issue when it comes to performing longitudinal studies on the model because it is hard to find pathogen-free Rhesus macaques.

RhCMV remains life-long in its Rhesus hosts and like HCMV, has evolved a plethora of mechanisms enabling it to antagonize, modulate, or evade its host immune response. Nearly 60% of RhCMV proteins are homologous to known HCMV proteins [95]. There are 135 out of 260 conserved open reading frames (ORFs) that include the structural, replicative and transcriptional regulatory proteins, and proteins involved in immune evasion [93], including cytokines, chemokines, and chemokine receptors. For instance, RhCMV encodes an ortholog of IL-10 just like HCMV does. RhcmvIL-10 is distinctive in that it has a 25% identity with cellular IL-10 proteins and the gene has three introns, compared to two introns for the UL111A gene in HCMV [96]. RhCMV IL-10 homologs can exert profound inhibition of pro-inflammatory cytokine production by human leukocytes and even alter MHC class I and class II expression [44], but more work is needed to determine if alternative splicing occurs and whether there are latency-associated forms of RhcmvIL-10.

So far, vaccine development has been the primary use of the rhesus model. One study found strong neutralizing antibody responses in a subset of macaques vaccinated with a modified version of RhcmvIL-10. There was reduced horizontal acquisition of RhCMV and significantly altered long-term immunity after those macaques became infected [94]. This result hints at the role of RhcmvIL-10 in establishing persistent or latent infections and suggests that immunization against cmvIL-10 could be a beneficial approach in HCMV vaccine development.

Another region where RhCMV has a similarity to HCMV is the US28 locus. In RhCMV, five tandem genes are positional homologs of US28, designated Rh214, Rh215, Rh216, Rh218, and Rh220 (formerly noted as RhUS28.1, RhUS28.2, RhUS28.3, RhUS28.4, and RhUS28.5 respectively) [97]. Of these, Rh214 and Rh220 are most closely related to US28, but all five have maintained the characteristics of seven-transmembrane proteins [97]. Like HCMV US28, Rh220 was found to bind the human chemokine fractalkine (CX_3_CL1) [97]. It remains to be seen whether Rh220, or any of the other US28 homologs, share other functions with US28, such as suppression of the MIEP to establish RhCMV latency. The Rhesus model has great potential to provide valuable insights for understanding latency and the development of effective vaccines and treatments for HCMV.

## 2. Concluding Remarks

Manipulation of the host immune response is a hallmark of HCMV, which establishes persistent infection and life-long latency in its host. HCMV gene products actively reprogram the initial pro-inflammatory response to infection, thwarting efforts to clear the virus and allowing for latency to be established in myeloid progenitor cells. HCMV also encodes proteins that imitate the structure and functions of host cytokines, chemokines, and chemokine receptors, and not only maintains a permissive environment but also contributes to the suppression of lytic infection. These viral orthologs create conditions that enable the establishment of latency. Overall, the modulation of cytokine signaling plays a critical role in HCMV infection.
